# Occurrence and human health risk assessment of antibiotics in cultured fish from 19 provinces in China

**DOI:** 10.3389/fcimb.2022.964283

**Published:** 2022-08-02

**Authors:** Yunyu Tang, Xiaoyi Lou, Guangxin Yang, Liangliang Tian, Yuan Wang, Xuanyun Huang

**Affiliations:** Key Laboratory of Control of Quality and Safety for Aquatic Products, Ministry of Agriculture and Rural Affairs, East China Sea Fisheries Research Institute, Chinese Academy of Fishery Sciences, Shanghai, China

**Keywords:** antibiotics, cultured fish, fluoroquinolones, tetracyclines, human health risk assessment

## Abstract

The occurrence of antibiotics and potential health risk of 300 cultured fish samples from 19 provinces in China were investigated. The levels of 28 antibiotics (15 fluoroquinolones, 4 tetracyclines, 8 macrolides and rifampin) in 8 fish species were measured through liquid chromatography electrospray tandem mass spectrometry. As a result, 10 antibiotics were detected with an overall detection frequency of 24.3%, and the individual detection frequency of antibiotics ranged from 0.33 to 16.7%. The extremely high concentrations (above 100 µg/kg) of doxycycline and erythromycin were found in the samples. Antibiotics with high detection frequency was noticed in largemouth bass (41.2%), followed by snakehead (34.4%) and bream (31.2%). Specifically, Heilongjiang, Xinjiang, Qinghai and Gansu presented high detection frequency values of more than 60%. Moreover, the highest mean concentration was observed in Shandong, and the concentration covered from 34.8 µg/kg to 410 µg/kg. Despite the high detection frequency and levels of antibiotics were found in samples, ingestion of cultured fish was not significantly related to human health risks in China, according to the calculated estimated daily intakes and hazard quotients. These results provided us the actual levels of antibiotics in cultured fish and human health risk assessment of consuming fishery products.

## Introduction

With the increase of human dietary demands, the aquatic products containing high protein, nutrition and special flavor have attracted wide attention of the world (Mishra, 2018). In the past three decades, aquaculture industry in China exhibited a rapidly increasing demand both in domestic and global markets. Till now, China produces more than one-third of the global fish supply (Cao et al, 2015; Wang et al., 2019). With over-exploited domestic fisheries, intensive and high-density culture is adopted to afford high yields and profits, while resulting in the increased fish diseases and mortalities (Mallory, 2013). Therefore, antibiotics are widely used for preventing and treating these diseases (Kümmerer, 2009; Hu and Cheng, 2016). However, excessive use of antibiotics will induce the antibiotics residues in fish and natural water environments, which may lead to the potential human health risk caused by the ultimate accumulation in humans *via* dietary consumption (Postigo and Richardson, 2014; Wang and Helbling, 2016; Urbano et al., 2017). Although the guidelines of antibiotics used in aquaculture were issued by a lot of countries, the improper sale and use of antibiotics were of frequent occurrence (Liu et al., 2017b). On the other hand, the antibiotics contained in fish were found from the other potential sources such as wastewater from agriculture areas, which can persist in the aquatic environment for a long time and transported and distributed easily *via* water systems (Alhaji et al., 2021). Additionally, it is noteworthy that many of antibiotics used in aquaculture are the same or similar to those for human use, such as tetracyclines, macrolides and rifampin. A long-term crossover of these antibiotics tended to reduce the effects for treating infectious diseases in humans (Zhou et al., 2021). As a result, it is necessary to investigate the occurrence and levels of antibiotics in the cultured fish.

Although the antibiotics residues in fish are at trace-level concentrations (Cui et al., 2018), long-term exposure may raise various human health concerns, including the enhanced antibiotics resistance, changes in metabolism and composition of gut microbiota (Wang et al., 2016; Caniça et al., 2019). Until now, the occurrence and levels in types of antibiotics and aquatic species were limited in the reported articles, which cannot reveal the antibiotics residues levels in whole aquaculture in China. Thus, a total of 28 antibiotics, including macrolides (e.g., erythromycin), tetracyclines (e.g., oxytetracycline), fluoroquinolones (e.g., enrofloxacin) and rifampin, were monitored in 300 cultured fish samples. The emphasis of this study was placed on the occurrence and levels of antibiotics in various fish species collected from 19 provinces in China. Moreover, the potential human health risks associated with antibiotics were evaluated based on the calculated estimated daily intake of the cultured fish. This study summarized the occurrence and levels of antibiotics in cultured fish in China and provided the human health risk assessment of consuming these fish.

## Materials and methods

### Chemicals and sample collections

High purity standards (> 97.5%) of 28 antibiotics were obtained from Dr Ehrenstorfer (Augsburg, Germany). The detailed information of 8 macrolides (MLs), 4 tetracyclines (TCs), 15 fluoroquinolones (FQs) and rifampin (RIF) were listed in the **Table S1**. Methanol, acetonitrile and formic acid were of chromatographic grade, purchased from Merck Ltd. (Whitehouse Station, USA). Ultra-pure water (18.2 MΩ cm quality or better) was obtained from a Milli-Q water purification system (Millipore, Bedford, USA). Stock solutions of each antibiotic were prepared in methanol at 500 µg/mL, and stored in the dark at −20°C.

**Figure 1:**
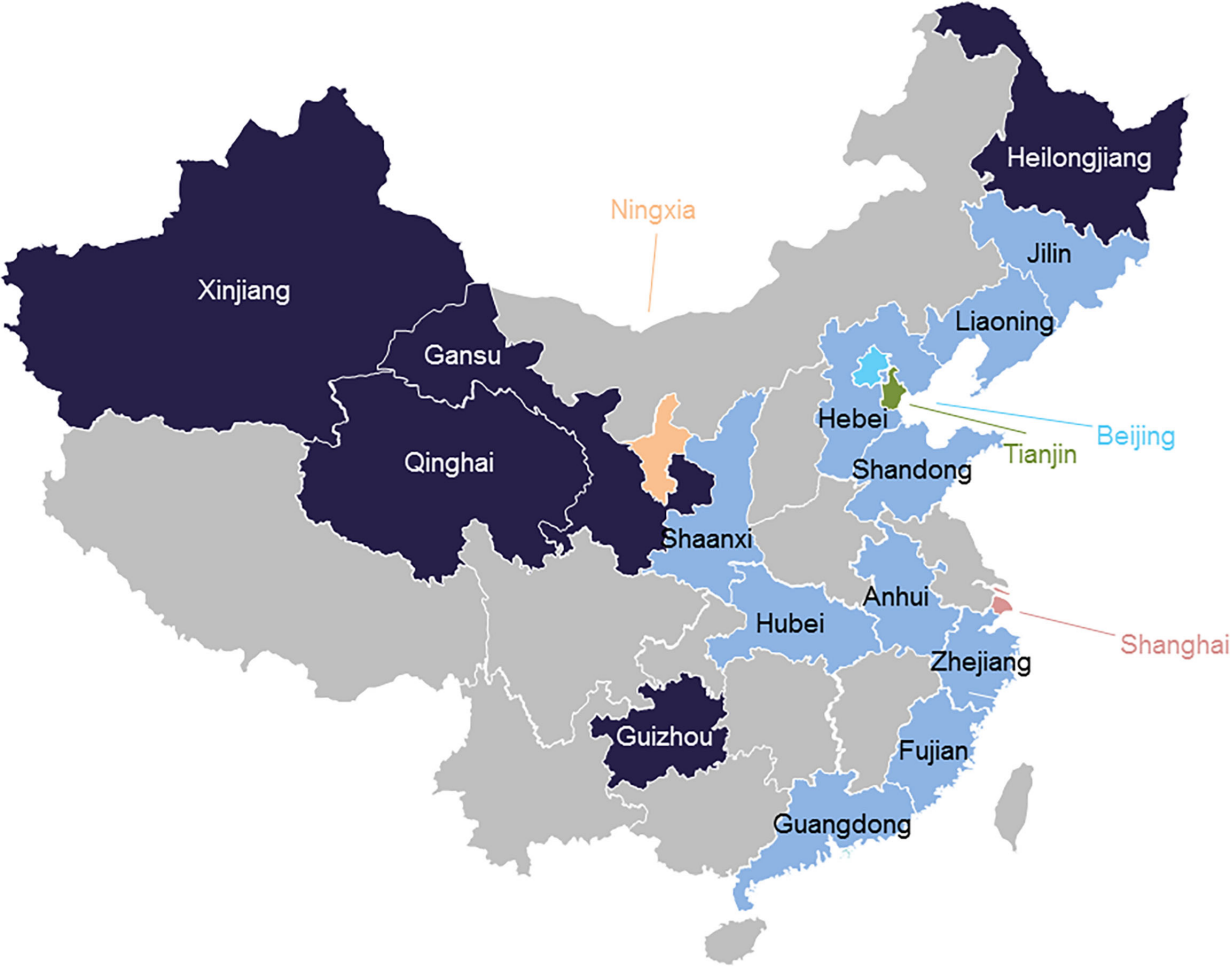
Location of sampling sites in China.

The 300 fish containing 8 species, including grass carp (*Ctenopharyngodon idella*), common carp (*Cyprinus carpio*), crucian carp (*Carassius carassius*), tilapia (*Oreochromis* sp.), bream (*Parabramis pekinensis*), largemouth bass (*Micropterus salmoides*), snakehead (*Channa argus*) and large yellow croaker (*Larimichthys crocea*) were collected from 19 provinces in China, including Anhui, Gansu, Shandong, Shaanxi, Ningxia, Zhejiang, Jilin, Hebei, Heilongjiang, Beijing, Guizhou, Tianjin, Liaoning, Xinjiang, Qinghai, Shanghai, Guangdong, Hubei and Fujian provinces (**Figure 1**). The proportions of samples in different provinces and species were shown in **Figures S1**, **S2**. Each sample contained 3–6 individuals in order to ensure the sample capacity. The alive fish were purchased from trading markets. Then the fish were stunned, dispatched and gutted by the butcher. After that, muscles of several individuals were sliced and combined into a bag as one sample. The samples were kept in a refrigerator at −20°C for 12 hours and transferred to the laboratory by airlift. In the lab, the samples were mixed, homogenized and stored at −20°C in the dark prior to the analysis.

### Sample preparation and instrumental analysis

The antibiotics extraction and purification methods were optimized according to the previous analytical method (Zhou et al., 2012). Briefly, 5 g (± 0.05 g) of sample were added with 0.5 mL of 0.1 mol/L EDTANa_2_ solution and 10 mL acetonitrile, followed by ultrasonication for 15 min. Subsequently, 5 g of anhydrous sodium sulfate was added and the samples were shaken by vortex mixing for 1 min. Then, the samples were centrifuged at 4500 r/min for 10 minutes, and the supernatant was transferred into a QuEChERS cube to degrease for purification. The purified liquids were centrifuged at 4500 rpm/min for 10 minutes. Finally, 3 mL of solution was combined with 1 mL water and filtered for analysis.

Antibiotics were detected by a Thermo Scientific HPLC system and a Thermo TSQ Quantum Ultra triple-quadrupole mass spectrometer with electrospray ionization (ESI) source (Thermo Fisher Scientific, Waltham, USA). Chromatographic separation was performed on an C_18_ column (100 mm × 4.6 mm, 2.7 μm) at 40°C. The mobile phase rate was 0.3 mL/min and the gradient elution program was described in **Table S2**. The antibiotics were detected by multiple reactions monitoring (MRM) mode with an electrospray ionization (ESI^+^) source and the injected volume was 10 μL. The spray voltage was 3700 V with 320°C of capillary temperature. The sheath gas and auxiliary gas flow rate were 30 psi and 15 psi, respectively. Details on antibiotic mass spectrometry conditions are available in **Table S3**. All the operation, data acquisition and analysis were controlled through the Thermo Xcalibur 3.1.

The details of the limits of detection (LOD) and quantitation (LOQ) for all antibiotics were listed in **Table S1**. The antibiotic residues in fish muscle showed a good linearity in a wide range of concentrations and corresponding correlation coefficient (R^2^) ranged from 0.990 to 0.999. The recoveries of antibiotics were in the range of 70%−115% with the relative standard deviations (RSD) belowing 15%.

### Daily exposure dose

With the aim to investigate the antibiotic residues for daily exposure doses in edible fish, the estimated daily intake (EDI) of individual antibiotic was calculated according to the following equation (Chen et al., 2020):


(1) $$\text{EDI}=\frac{\text{C}\times \text{CI}}{\text{BW}}$$

where EDI (μg/(kg bw d)) is the estimated daily intake of the antibiotic in fish; C (μg/kg) means the concentration of the antibiotics detected in fish samples; CI (kg/day) is the daily intake of aquatic products (0.056 kg in the Fifth China Total Diet Study); BW (kg) stands for the body weight of the consumer (60 kg, average adult weight).

### Health risk assessment

Hazard quotients (HQ) is calculated as the ratio of EDI to acceptable daily intake (ADI), indicating the risk of individual antibiotic. In addition, hazard index (HI) is used to reflect cumulative health risks for total selected antibiotics. The HI is calculated as the sum of HQ, showing the cumulative risk of total antibiotics in samples. The formulas are calculated as follows:


(2) $$\text{HQ}=\frac{EDI}{ADI};\;HI=\int HQ$$

The ADI values are available from the literature or the authorities, as detailed in **Table S1**. In general, HQ ≥ 1 indicates a high risk to health, while HQ < 1 indicates a tolerable daily intake dose (Fang et al., 2021).

### Statistical analysis

The detection frequency and mean concentration of antibiotics were calculated. The samples of which antibiotics concentration below the limit of detection (LOD) were not counted for detection frequency. The values of antibiotic lower than the LOD were replaced with those of LOD/2 when the EDI was calculated (Zeng et al., 2020).

## Results and discussion

### Detection concentration and frequency of antibiotics

**Figure 2:**
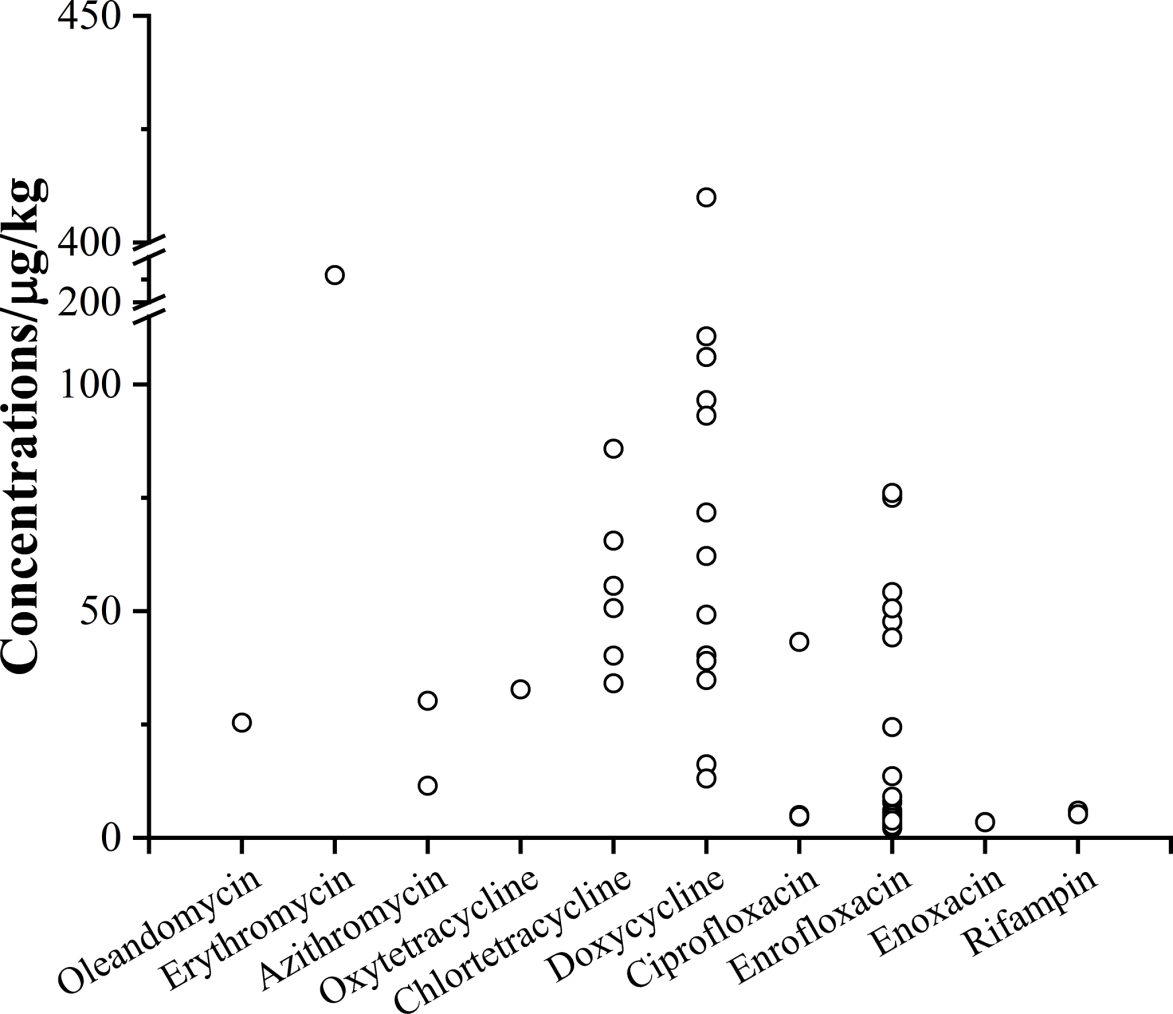
Concentrations of antibiotics in the positive samples.

Aquatic products provide abundant nutrition and protein to human consumption, which have been a part of daily diet. To assure the food safety, it is crucial to continuously monitor the occurrence and levels of antibiotics in aquatic products (Aubourg, 2018; Ni et al., 2021). Therefore, we determined the residues of 28 antibiotics in 8 consumable fish species from 19 provinces in China. The antibiotics with concentration below LOD were not listed in **Table 1**. As shown in **Table 1**, an overall detection frequency of antibiotics was 24.3%, and the individual antibiotic frequency ranged from 0.33% to 16.7%. Among 4 antibiotic classes, the FQs were detected in the samples with the highest detection frequency of 16.3%. This observation was consistent with previous investigations that the FQs were frequently detected in cultured aquatic products, indicating their common occurrence in the commercial fish (He et al., 2016; Liu et al., 2022). Consideration of the ciprofloxacin as the main metabolite of enrofloxacin (Xu et al., 2006; Elezz et al., 2019), the sum of the detection frequency for ciprofloxacin and enrofloxacin was calculated to evaluate the sample. As a result, 98% of the positive samples were detected containing enrofloxacin, ciprofloxacin or both antibiotics, with the concentration ranging from 2.17 µg/kg to 90.8 µg/kg (**Figure 2**), which were lower than the maximum residue limit (MRL) of 100 µg/kg for the sum of the concentration for these two antibiotics. As shown in **Figure S3**, the sum of the concentration for enrofloxacin and ciprofloxacin lied during 1.0–10.0 μg/kg at the ratio of 77.6%. Similar observations were also reported in sediment (Chen et al., 2015), water (Liang et al., 2013) and the other organism (Li et al., 2012; Zhang et al., 2021). These results indicated that there may be several possible reasons for the high frequency of enrofloxacin, such as the predominant antibiotics used for cultured fish (Ministry of Agriculture, 2007), the antibiotics contamination of the environment (Xu et al., 2018) and other additive (health care products, organic fertilizers and/or vitamin additives) (Song et al., 2017; Li et al., 2018).

**Table 1:** Detection frequency of antibiotics in different fish species.

Antibiotics	% (N)^a^(n=300)	grass carp	common carp	crucian carp	tilapia	bream	largemouth bass	snakehead	large yellow croaker
Oleandomycin	0.33 (1)	–	–	–	–	–	–	–	2.38 (1)
Erythromycin	0.33 (1)	–	–	–	–	–	–	–	2.38 (1)
Azithromycin	0.67 (2)	–	–	–	–	–	11.8 (2)	–	–
**Macrolides**^b^	**1.00 (3)**	–	–	–	–	–	**11.8 (2)**	–	**4.76 (2)**
Oxytetracycline	0.33 (1)	–	–	–	–	–	–	–	2.38 (1)
Chlortetracycline	2.00 (6)	–	6.52 (3)	2.38 (1)	7.41 (2)	–	–	–	–
Doxycycline	4.33 (13)	1.61 (1)	13.0 (6)	2.38 (1)	–	6.25 (2)	–	9.38 (3)	–
**Tetracyclines**^b^	**6.33 (19)**	**1.61 (1)**	**19.6 (9)**	**4.76 (2)**	**7.41 (2)**	**6.25 (2)**	**-**	**9.38 (3)**	–
Ciprofloxacin	1.33 (4)	–	2.17 (1)	–	–	3.12 (1)	–	6.25 (2)	–
Enrofloxacin	16.7 (48)	16.1(10)	10.9 (5)	9.52 (4)	18.5 (5)	21.9 (7)	29.4 (5)	25.0 (8)	9.52 (4)
Enoxacin	0.67 (2)	–	2.17 (1)	–	–	–	–	3.12 (1)	–
**Fuoroquinolones**^b^	**16.3 (49)**	**16.1 (10)**	**13.0 (6)**	**9.52 (4)**	**18.5 (5)**	**21.9 (7)**	**29.4 (5)**	**25.0 (8)**	**9.52 (4)**
Rifampin	0.67 (2)	1.61 (1)	–	–	–	3.12 (1)	–	–	–
**Total**	**24.3 (73)**	**19.4 (12)**	**30.4 (14)**	**14.3 (6)**	**25.9 (7)**	**31.2 (10)**	**41.2 (7)**	**34.4 (11)**	**14.3 (6)**

a % (N), detection frequency, % (the number of positive detection). -, < limits of detection (LODs).

b Sum of frequency in corresponding category for individual.

The detection frequency of TCs was 6.33% in this study. Among 4 monitored TCs, doxycycline was the dominant antibiotic, accounting for 4.33% of the detection frequency. It is noteworthy that 53.8% of samples detected on TCs presented high concentrations of doxycycline above 50 µg/kg (**Figure 2**). Moreover, three samples showed relatively high concentrations of 106 µg/kg, 111 µg/kg and 410 µg/kg on doxycycline, which were higher than the MRL of 100 µg/kg. Generally, the antibiotic concentration in commercial fish was below the MRL, but few exceptions exceeding the limits were reported (Kang et al., 2018; Liu et al., 2018). Additionally, Griboff et al. (2020) reported that the MRL for doxycycline was exceeded in 44% of the fish samples from supermarkets and aquaculture farms in Argentina. These observations may be ascribed that doxycycline was frequently used during the growth and adult stages of fish, leading to the high concentration of doxycycline in culture water, which resulted in the bioaccumulation in cultured fish (Zhang et al., 2021). Compared with the doxycycline, oxytetracycline (0.33%) and chlortetracycline (2.00%) showed low detection frequency, with the concentrations in the range of 32.8−85.8 µg/kg. However, high levels of oxytetracycline and chlortetracycline were found in USA (Done and Halden, 2015), Spain (Grande-Martínez et al., 2018) and Nigeria (Alhaji et al., 2021). Griboff et al. (2020) reported that the extensive use of TCs would lead to antibiotic resistance by some bacterial species in aquaculture.

The MLs are commonly used as feed additives in fish cultivation for growth promotion and disease prevention and treatment (Zhao et al., 2010; Liu et al., 2017b). MLs accounted for a minor detection frequency of 1.00% in all samples. Among the 8 MLs, oleandomycin, erythromycin and azithromycin were detected in 0.33%, 0.33% and 0.67% of the samples, respectively. It should be noted that only one sample was detected on erythromycin, but its concentration was up to 206 µg/kg, together with the concentration of 25.4 µg/kg on oleandomycin. Two samples showed the concentrations of 30.2 µg/kg and 11.5 µg/kg on azithromycin (**Figure 2**), respectively. In this regard, Chen et al. (2015) reported that erythromycin was the main antibiotic with the concentrations ranging from 2498 to 15,090 µg/kg in the adult *Fenneropenaeus penicillatus* from Hailing Island. However, much lower concentrations of 3.5–12 µg/kg were detected in cultured fish from other typical aquaculture (Chen et al., 2018). These observations indicated that the large difference of concentrations in aquatic products might be ascribed to the different potential source of MLs, including disease treatment, residues in sediments and/or water of aquaculture ponds and feeds additives (Zhang et al., 2023).

Rifampin is widely used to treat bacterial infections and tuberculosis as a human antibiotic (Lu et al., 2009; Seung et al., 2004). Besides, rifampin can prevent the RNA production by bacteria to resist vaccinia virus (Norton and Holland, 2012; Charity et al., 2007). It is reported that rifampin can effectively control the bacterial diseases of fish, such as edwardsiellosis and columnaris disease (Olivares-Fuster & Arias, 2011). At present, there are no relevant policies, regulations and standards for its use in China. However, it is found that rifampicin is being used in aquaculture (Li et al., 2020). It is noteworthy that rifampin can easily enter the edible tissues of aquatic products due to the fat soluble characters, resulting in accumulations in human body, which tends to promote the antibiotic resistant bacteria (Rico et al., 2012) and health risk to human, such as the skin discoloration, pruritus, nausea or vomiting (Zaki et al., 2013). Among 300 samples, a frequency detection of rifampin was just 0.67% with the concentration of 5.2 µg/kg and 6 µg/kg, respectively. In regard to such low frequency detection, it could be considered little health risk associated with rifampin residues.

### Concentration and detection frequency of fish species

**Figure 3:**
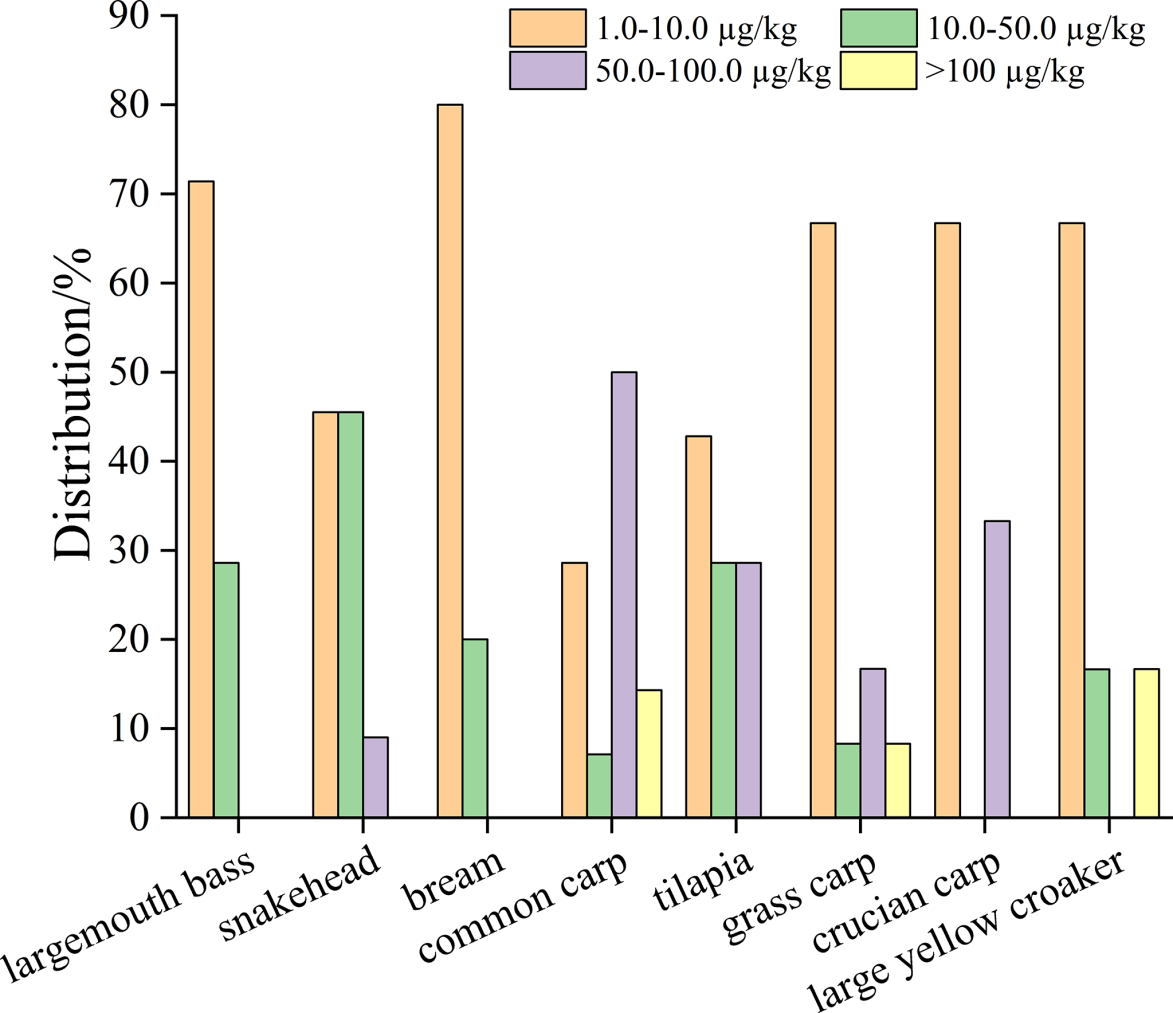
Distributions of concentration in different aquatic species.

Overall, at least one antibiotic was tested positive in each fish species. As shown in **Table 1**, the detection frequency of antibiotics in fish species was all higher than 10%. The largemouth bass showed the highest detection frequency of 41.2%, resulting from 29.4% of FQs and 11.8% of MLs. However, the antibiotic concentrations of largemouth bass were just detected in the ranges of 2.77−30.2 µg/kg (**Figure S4**). The detection frequency of 34.4% was found in snakehead, with 25.0% of FQs and 9.38% of TCs detection. The distributions of the positive snakehead samples were 45.5%, 45.5% and 9.00% in the concentration ranges of 1.0−10.0 µg/kg, 10.0−50.0 µg/kg and 50.0−100.0 µg/kg (**Figure 3**), respectively. Similar detection frequency was observed in bream and common carp for 31.2% and 30.4%, respectively. However, 50% of the detected concentration values appeared in the range of 50.0−100.0 µg/kg for common carp. Among the whole fish species, the tilapia, grass carp, crucian carp and large yellow croaker showed lower detection frequency of 25.9%, 19.4%, 14.3% and 14.3%, respectively. Whereas, high concentration of 410 µg/kg on TCs was detected for grass carp, and 231 µg/kg on MLs for large yellow croaker. In terms of rifampin, the detection frequency of 1.61% and 3.12% were noticed for grass carp and bream, respectively.

It is noteworthy that largemouth bass had the highest detection frequency than those of other fish species due to the severe microbial infectious diseases, leading to huge economic loss in largemouth bass farming (Deng et al., 2011; Ma et al., 2013). The lowest detection frequency occurred in crucian carp and large yellow croaker, which was mainly attributed to the low frequency of FQs. The FQs were detected in all fish species and showed relatively higher detection frequency than any other antibiotics. This observation indicated that the consumption of FQs in fish farm was relatively high in China (Zhang et al., 2012) and FQs were used in various fish species (Liu et al., 2018). Gao et al. (2012) reported that the actual maximum used enrofloxacin was mainly acted as feed additives in aquaculture among the FQs. Amidst all of fish species, common carp had high detection frequency (19.6%) and concentration (34.8−111 µg/kg) of TCs in this study, although high concentrations of TCs manly appeared in benthic fish species (Liu et al., 2018). Types and species of aquatic fish and/or aquaculture environments may serve as an explanation for the different detection frequency of the antibiotics in cultured fishes.

As shown in **Figure 3**, the concentrations of positive samples were mainly located in the region of 1.0−10.0 µg/kg for the majority of fish species. The common carp samples contributed the high occurrence and levels of antibiotics contamination, showing the proportion of 50% in 50.0−100.0 µg/kg and 14.3% over 100 µg/kg. Moreover, the concentrations beyond 100 µg/kg were also detected in grass carp and large yellow croaker with 8.3% and 16.7% of positive samples, respectively. These observations indicated that fish species afforded an effect on the antibiotic distribution. Meanwhile, the concentrations differed among various aquatic species might be associated with the different antibiotics bioaccumulation.

### Concentration and detection frequency of provinces

The detailed antibiotic detection frequency of provinces is summarized in **Table 2**. Anhui, Shaanxi, Ningxia and Fujian provinces were not listed due to no antibiotics detection in these provinces. The highest detection frequency of antibiotics was 66.7% obtained from Heilongjiang (**Figure S5**), with the concentrations ranging from 2.64 µg/kg to 85.8 µg/kg (**Table 3**). The total detection frequency in Xinjiang, Qinghai, Guizhou and Gansu were 64.7%, 62.5%, 60.0% and 55.6% respectively. Among above five provinces, Heilongjiang also showed the highest mean concentration of 83.5 µg/kg, followed by 74.7 µg/kg in Qinghai (**Table S5**). The rest of three provinces owned similar mean concentrations of antibiotics in the range of 55.1−59.6 µg/kg. Shandong had 36.0% of positive samples totally detected on TCs, with the highest mean concentration of 88.2 µg/kg TCs among overall 19 provinces, containing the high concentrations of 111 µg/kg, 157 µg/kg and 410 µg/kg (**Figure S5**). Shanghai presented the low detection frequency of 6.67% with the mean concentration of 52.3 µg/kg. The detection frequency of 10.0−33.3% was found in other provinces, and the mean concentrations lied in the range of 52.6–66.0 μg/kg.

**Table 2:** Detection frequency of antibiotics in relation to province.

Sites	% (N)^a^	Macrolides (%/n)	Tetracyclines (%/n)	Fuoroquinolones (%/n)	Rifampicin (%/n)
Beijing	**33.3 (4)**	–	16.7 (2)	16.7 (2)	–
Shandong	**36.0 (9)**	–	36.0 (9)	–	–
Zhejiang	**20.8 (5)**	–	4.17 (1)	16.7 (4)	–
Jilin	**15.4 (2)**	–	–	15.4 (2)	–
Hebei	**18.8 (3)**	–	–	18.8 (3)	–
Heilongjiang	**66.7 (6)**	–	33.3 (3)	33.3 (3)	–
Gansu	**55.6 (5)**	–	–	55.6 (5)	–
Guizhou	**60.0 (3)**	–	20.0 (1)	40.0 (2)	–
Tianjin	**11.1 (1)**	–	–	11.1 (1)	–
Liaoning	**20.0 (8)**	–	–	20.0 (8)	–
Xinjiang	**64.7 (11)**	–	–	58.8 (10)	5.88 (1)
Qinghai	**62.5 (5)**	25.0 (2)	–	37.5 (3)	–
Shanghai	**6.67 (2)**	–	6.67 (2)	–	–
Guangdong	**26.7 (8)**	3.33 (1)	3.33 (1)	16.7 (5)	3.33 (1)
Hubei	**10.0 (1)**	–	–	10.0 (1)	–

a % (N), sum of frequency in provinces, % (the number of positive detection). -, < limits of detection (LODs).

**Table 3:** Concentrations of antibiotics in different provinces.

Sites	N (n)^a^	Range (µg/kg)^b^	Mean (µg/kg)^c^
Beijing	12 (4)	3.54 − 96.6	64.4
Shandong	25 (9)	34.8 − 410	88.2
Zhejiang	24 (5)	2.39 – 32.8	53.3
Jilin	13 (2)	2.59 – 75.8	58.0
Hebei	16 (3)	3.11 – 8.04	52.9
Heilongjiang	9 (6)	2.64 −85.8	83.5
Gansu	9 (5)	4.73 – 24.4	57.6
Guizhou	5 (3)	2.89 – 40.2	59.6
Tianjin	9 (1)	5.61	52.6
Liaoning	40 (8)	2.63 – 5.72	52.7
Xinjiang	17 (11)	2.72 – 10.9	55.1
Qinghai	8 (5)	2.17 – 90.8	74.7
Shanghai	30 (2)	13.1 – 16.2	52.3
Guangdong	30 (8)	2.70 – 76.1	66.0
Hubei	10 (1)	9.06	52.9

a N, the number of sample in sites; (n) the number of positive sample.

b The concentration for positive detection.

c Antibiotic concentrations below LOD were replaced with those of LOD/2.

Comparing with those in typical aquaculture provinces, such as Guangdong, Hubei and Fujian, the detection frequency of antibiotics in provinces located in the regions where were a great distance away from the aquatic farmed areas (**Figure 1**), were obviously higher (55.6−66.7%), including Heilongjiang, Gansu, Guizhou, Xinjiang and Qinghai. This observation may be rationalized by the fact that the antibiotics were applied during the transportation to ensure the survival rate of the fish. Moreover, as mentioned above, the high concentrations mainly occurred in Shandong. The concentration of 33.3% of positive samples exceeded 100 µg/kg in Shandong and 12.5% in Guangdong (**Figure S5**), which may be ascribed to significant effects of aquaculture environment and drug-using habits in fish farmed areas (Kang et al., 2018; Zhang et al., 2021). These results suggested that the fish with illegal antibiotics use and/or insufficient withdrawal time were marketed in these regions (Menkem et al., 2019).

The FQs were observed in almost all provinces except for Shandong and Shanghai, and the mean concentration was in the range of 7.5 μg/kg to 25.1 μg/kg (**Table S5**). The TCs were detected in 7 provinces and the mean concentration in the range of 40.3−76.2 μg/kg. MLs were just found in Qinghai and Guangdong, with the mean concentration of 9.09 μg/kg and 11.7 μg/kg, respectively. RIF just accounted for a minor proportion of 5.88% and 3.33% in Xinjiang and Guangdong, respectively. Compared with previous research, the levels of FQs in this study were obviously lower than those of samples from Liao River, Haihe River and Baiyang Lake (Bai et al., 2014; Liu et al., 2017a; Chen et al., 2018), while the mean concentrations of TCs were comparable to those of farmed fish in Dalian and Hubei in China (Liu et al., 2013; Liu et al., 2018), Spain (Chafer-Pericas et al., 2011), and rainbow trout in Iran (Barani and Fallah, 2015), but it was much higher than those in Liao River Bay (Bai et al., 2014). These observations suggested that the different concentration of antibiotics may be associated with the different pollution levels in these regions.

### Estimated daily intake and health risk assessment

Consumption of antibiotic-contaminated aquatic products may cause potential risks to human health. The MRL was set for the antibiotic residues in animal food to assess the health risk for human consumption (Ministry of Agriculture, 2019). Fish consumption would pose a potential health risk when the values of antibiotic residues in edible fish were higher than its MRL. The MRLs for doxycycline and erythromycin in fish were 100 and 200 µg/kg wet wt, respectively. In the present study, the high concentrations of doxycycline were 106 µg/kg and 111 µg/kg in common carp, 410 µg/kg in grass carp and 206 µg/kg of erythromycin in large yellow croaker. These results indicated that consumption of these contaminated fish might pose health risks to humans.

Additionally, the potential human health risks should also be evaluated based on the dietary exposure of these antibiotics. In this respect, hazard quotient (HQ) was calculated by comparing the estimated daily intake (EDI) with the acceptable daily intake (ADI) established by WHO to assess the potential health risks associated with human consumption of contaminated fish (Boonsaner and Hawker, 2013; Liu et al., 2017b). Hence, the EDI and HQ were calculated to evaluate the health risk assessment of cultured fish in various aquatic species and provinces in this study. The calculated EDI values of the individual antibiotics ranged from 4.9×10^-4^ to 1.3×10^-2^ μg/(kg bw d) (**Table S6**). Although the detection frequency of FQs was the highest, the calculated HQ was just 4.5×10^-3^ due to the relatively low concentrations (**Table 4**). The highest EDI value was 0.041 μg/(kg bw d) obtained in TCs, which might be ascribed to the several high concentration values detected on doxycycline. As a result, the HQ value of TCs was 8.3×10^-3^, suggesting no direct detrimental effects from consumption of TCs-contaminated fish because the HQ value was much lower than 1. It is noteworthy that the HI values of four antibiotics classes were less than 1, indicating that no significant risk was associated with overall antibiotics residues from the ingestion of cultured fish for human health.

**Table 4:** HQ of 28 antibiotics based on the estimated daily exposure dose per aquatic species.

Antibiotics	HQ×10^-2^	grass carp	common carp	crucian carp	tilapia	bream	largemouth bass	snakehead	large yellow croaker
Oleandomycin	0.011	–	–	–	–	–	–	–	0.020
Erythromycin	0.022	–	–	–	–	–	–	–	0.10
Azithromycin	0.012	–	–	–	–	–	0.054	–	–
**Macrolides** ^a^	**0.091**	**0.075**	**0.075**	**0.075**	**0.075**	**0.075**	**0.12**	**0.075**	**0.18**
Oxytetracycline	0.19	–	–	–	–	–	–	–	0.20
Chlortetracycline	0.20	–	0.26	0.21	0.22	–	–	–	–
Doxycycline	0.25	0.31	0.37	0.21	–	0.23	–	0.21	–
**Tetracyclines** ^a^	**0.83**	**0.87**	**1.00**	**0.79**	**0.78**	**0.79**	**0.75**	**0.77**	**0.76**
Ciprofloxacin	0.032	–	0.028	–	–	0.029	–	0.092	–
Enrofloxacin	0.12	0.14	0.16	0.036	0.31	0.066	0.061	0.14	0.039
Enoxacin	0.024	–	0.026	–	–	–	–	0.028	–
**Fuoroquinolones** ^a^	**0.45**	**0.46**	**0.49**	**0.36**	**0.63**	**0.40**	**0.39**	**0.54**	**0.37**
Rifampin	5.0E-06	5.38E-06	4.67E-06	4.67E-06	4.67E-06	6.25E-06	4.67E-06	4.67E-06	4.67E-06
**Total** ^b^	**1.37**	**1.40**	**1.57**	**1.23**	**1.49**	**1.26**	**1.25**	**1.39**	**1.30**

a Sum of hazard quotient of antibiotics in corresponding category for individual.

b The sum of the HQ for individual antibiotic; -, < limits of detection (LODs).

Among the different fish species, the EDI values of total antibiotics ranged from 0.051−0.057 μg/(kg bw d) besides common carp. The estimated daily exposures to TCs and FQs in common carp were considerably higher than those in the other species. As a result, the highest EDI and HQ values of 0.064 μg/(kg bw d) and 1.57×10^-2^ were found in common carp, suggesting that ingestion of common carp would pose higher health risk than any other fish species. Considering that HQ values of various fish species were all lower than 1, showing that the potential risk to human health was relatively low by consumption of fish purchased from market. Similar risk assessment for cultured fish was observed in many other researches (Bercu et al., 2008; Varol and Sünbül, 2019; Zhou et al., 2020; Ye et al., 2021). On the other hand, the HI values of Heilongjiang and Qinghai were 2.09×10^-2^ and 2.06×10^-2^, respectively, which were relatively higher than those of 1.18×10^-2^−1.84×10^-2^ in other provinces (**Table S7**). It can be considered that consuming fish from these areas would not pose a health risk to humans. However, it needs to be pointed out that the human health risk assessment of the antagonistic or synergistic relationship among various antibiotics should be further investigated (Kümmerer, 2009). Moreover, the toxicity of the metabolites and transformation were not considered in this risk assessment (Cleuvers, 2004; Lu et al., 2020). As a result, a larger-scale monitoring and more comprehensive risk assessment are necessary to assess the risks related to dietary exposure for human health in the future work.

In conclusion, the occurrence and levels of 28 antibiotics were monitored in 300 fish samples collected from 19 provinces in China. The overall detection frequency of antibiotics was 24.3%. The highest detection frequency of 16.3% was for FQs and 41.2% for largemouth bass. Moreover, the high detection frequency of more than 60% in Heilongjiang, Xinjiang, Qinghai and Gansu. The highest mean concentration was noticed in Shandong, and the concentration covered from 34.8 µg/kg to 410 µg/kg. Thus, it is necessary to do further investigation on larger-scale and comprehensive risk assessment research to understand the condition of antibiotic residues in aquatic products and prevent adverse effects that may be caused by these antibiotics. In addition, compared with the ADI proposed by WHO, the calculated EDI values of antibiotics in various fish species and provinces were very low, indicating no direct detrimental effects related to consuming cultured fish for human health in China. These results provided us the actual occurrence and levels of antibiotics in cultured fish and human health risk assessment of consuming fishery products.

## Data availability statement

The original contributions presented in the study are included in the article/**Supplementary Material**. Further inquiries can be directed to the corresponding author.

## Ethics Statement

The animal study was reviewed and approved by Animal Research Committees of the East China Sea Fisheries Research Institute.

## Author contributions

YT wrote the manuscript. YT, XL, and XH participated in the study conception and design. YT, GY, LT, and YW performed the experiments. XL, YT, and LT performed the statistical analysis. YT, YW, and XH revised the manuscript. All authors contributed to the article and approved the submitted version.

## Funding

This study was financially supported by Central Public-interest Scientific Institution Basal Research Fund, ECSFR, CAFS (2018T02) and the Project of National Agricultural Product Quality and Safety Risk Assessment (GJFP201600901).

## Conflict of Interest

The authors declare that the research was conducted in the absence of any commercial or financial relationships that could be construed as a potential conflict of interest.

## Publisher’s note

All claims expressed in this article are solely those of the authors and do not necessarily represent those of their affiliated organizations, or those of the publisher, the editors and the reviewers. Any product that may be evaluated in this article, or claim that may be made by its manufacturer, is not guaranteed or endorsed by the publisher.

## References

[B1] Alhaji N. B. Maikai B. V. Kwaga J. K. P. (2021). Antimicrobial use, residue and resistance dissemination in freshwater fish farms of north-central Nigeria: One health implications. Food Contr. 130, 108238. doi: 10.1016/j.foodcont.2021.108238

[B2] Aubourg S. P. (2018). Impact of high-pressure processing on chemical constituents and nutritional properties in aquatic foods: a review. Int. J. Food Sci. Tech. 53, 873–891. doi: 10.1111/ijfs.13693

[B3] Bai Y. W. Meng W. Xu J. Zhang Y. Guo C. S. (2014). Occurrence, distribution and bioaccumulation of antibiotics in the liao river basin in China. Environ. Sci-Proc. Imp. 16, 586–593. doi: 10.1039/c3em00567d 24509869

[B4] Barani A. Fallah A. A. (2015). Occurrence of tetracyclines, sulfonamides, fluoroquinolones and florfenicol in farmed rainbow trout in Iran. Food Agr. Immunol. 26, 420–429. doi: 10.1080/09540105.2014.950199

[B5] Bercu J. P. Parke N. J. Fiori J. M. Meyerhoff R. D. (2008). Human health risk assessments for three neuropharmaceutical compounds in surface waters. Regul. Toxicol. Pharmacol. 50, 420–427. doi: 10.1016/j.yrtph.2008.01.014 18331773

[B6] Boonsaner M. Hawker D. W. (2013). Evaluation of food chain transfer of the antibiotic oxytetracycline and human risk assessment. Chemosphere 93, 1009–1014. doi: 10.1016/j.chemosphere.2013.05.070 23790827

[B7] Caniça M. Manageiro V. Abriouel H. Moran-Gilad J. Franz C. M. (2019). Antibiotic resistance in foodborne bacteria. Trends Food Sci. Tech. 84, 41–44. doi: 10.1016/j.tifs.2018.08.001

[B8] Cao L. Naylor R. Henriksson P. Leadbitter D. Metian M. Troell M.. (2015). China’s aquaculture and the world’s wild fisheries. Science 347, 133–135. doi: 10.1126/science.1260149 25574011

[B9] Chafer-Pericas C. Maquieira A. Puchades R. Miralles J. Moreno A. (2011). Multiresidue determination of antibiotics in feed and fish samples for food safety evaluation. comparison of immunoassay vs LC-MS-MS. Food Contr. 22, 993–999. doi: 10.1016/j.foodcont.2010.12.008

[B10] Charity J. C. Katz E. Moss B. (2007). Amino acid substitutions at multiple sites within the vaccinia virus D13 scaffold protein confer resistance to rifampicin. Virology 359, 227–232. doi: 10.1016/j.virol.2006.09.031 17055024 PMC1817899

[B11] Chen L. Li H. Liu Y. Li Y. Yang Z. (2020). Occurrence and human health risks of twenty-eight common antibiotics in wild freshwater products from the xiangjiang river and comparison with the farmed samples from local markets. Food Addit. Contam. A. 37, 770–782. doi: 10.1080/19440049.2020.1730987 32174263

[B12] Chen H. Liu S. Xu X. R. Diao Z. H. Sun K. F. Hao Q. W.. (2018). Tissue distribution, bioaccumulation characteristics and health risk of antibiotics in cultured fish from a typical aquaculture area. J. Hazard. Mater. 343, 140–148. doi: 10.1016/j.jhazmat.2017.09.017 28946134

[B13] Chen H. Liu S. Xu X. Liu S. Zhou G. Sun K.. (2015). Antibiotics in typical marine aquaculture farms surrounding hailing island, south China: Occurrence, bioaccumulation and human dietary exposure. Mar. pollut. Bull. 90, 181–187. doi: 10.1016/j.marpolbul.2014.10.053 25467872

[B14] Cleuvers M. (2004). Mixture toxicity of the anti-inflammatory drugs diclofenac, ibuprofen, naproxen, and acetylsalicylic acid. Ecotox. Environ. Safe. 59, 309–315. doi: 10.1016/S0147-6513(03)00141-6 15388270

[B15] Cui C. Han Q. Jiang L. Ma L. Zhang T. (2018). Occurrence, distribution, and seasonal variation of antibiotics in an artificial water source reservoir in the Yangtze river delta, East China. Environ. Sci. pollut. R. 25, 1–10. doi: 10.1007/s11356-018-2124-x 29728969

[B16] Deng G. Li S. Xie J. Bai J. Chen K. Ma D.. (2011). Characterization of a ranavirus isolated from cultured largemouth bass (*Micropterus salmoides*) in China. Aquaculture 312, 198–204. doi: 10.1016/j.aquaculture

[B17] Done H. Y. Halden R. U. (2015). Reconnaissance of 47 antibiotics and associated microbial risks in seafood sold in the united states. J. Hazard. Mater. 282, 10–17. doi: 10.1016/j.jhazmat.2014.08.075 25449970 PMC4254636

[B18] Elezz A. Easa A. Atia F. Ahmed T. (2019). The potential impact data of tylosin and enrofloxacin veterinary antibiotics on germination and accumulation in barley seed as a forage crop and good dietary sources using LC/MS-MS. Data Brief 25, 1–7. doi: 10.1016/j.dib.2019.104326 PMC6700488 31453299

[B19] Fang L. Huang Z. Fan L. Hu G. Qiu L. Song C.. (2021). Health risks associated with sulfonamide and quinolone residues in cultured Chinese mitten crab (*Eriocheir sinensis*) in China. Mar. Pollut. Bull. 165, 112184. doi: 10.1016/j.marpolbul.2021.112184 33621905

[B20] Gao L. Shi Y. Li W. Liu J. Cai Y. (2012). Occurrence, distribution and bioaccumulation of antibiotics in the haihe river in China. J. Environ. Monitor. 14, 1248–1255. doi: 10.1039/c2em10916f 22402740

[B21] Grande-Martínez A. Moreno-Gonzalez D. Arrebola-Liebanas F. J. Garrido-Frenich A. Garcia-Campana A. M. (2018). Optimization of a modified QuEChERS method for the determination of tetracyclines in fish muscle by UHPLC-MS/MS. J. Pharmaceut. Biomed. 155, 27–32. doi: 10.1016/j.jpba.2018.03.029 29602055

[B22] Griboff J. Carrizo J. C. Bonansea R. I. Valdés M. E. Wunderlin D. A. Amé M. V. (2020). Multiantibiotic residues in commercial fish from argentina. the presence of mixtures of antibiotics in edible fish, a challenge to health risk assessment. Food Chem. 332, 127380. doi: 10.1016/j.foodchem.2020.127380 32603916

[B23] He X. Deng M. Wang Q. Yang Y. Yang Y. Nie X. (2016). Residues and health risk assessment of quinolones and sulfonamides in cultured fish from pearl river delta, China. Aquaculture 458, 38–46. doi: 10.1016/j.aquaculture.2016.02.006

[B24] Hu Y. Cheng H. (2016). Health risk from veterinary antimicrobial use in china’s food animal production and its reduction. Environ. Pollut. 219, 993–997. doi: 10.1016/j.envpol.2016.04.099 27180067

[B25] Kang H. Lee S. Shin D. Jeong J. Hong J. Rhee G. (2018). Occurrence of veterinary drug residues in farmed fishery products in south Korea. Food Contr. 85, 57–65. doi: 10.1016/j.foodcont.2017.09.019

[B26] Kümmerer K. (2009). Antibiotics in the aquatic environment–a review–part I. Chemosphere 75, 417–434. doi: 10.1016/j.chemosphere.2008.11.086 19185900

[B27] Liang X. Chen B. Nie X. Shi Z. Huang X. Li X. (2013). The distribution and partitioning of common antibiotics in water and sediment of the pearl river estuary, south China. Chemosphere 92, 1410–1416. doi: 10.1016/j.chemosphere.2013.03.044 23628172

[B28] Li Q. Cao J. Han G. Liu H. Yan J. Wu L.. (2020). Quantitative determination of rifampicin in aquatic products by stable isotope-dilution high liquid chromatography–tandem mass spectrometry. Biomed. Chromatogr. 34, e4810. doi: 10.1002/bmc.4810 32043607

[B29] Li Q. Na G. Zhang L. Lu Z. Gao H. Li R.. (2018). Effects of corresponding and non-corresponding contaminants on the fate of sulfonamide and quinolone resistance genes in the laizhou bay, China. Mar. pollut. Bull. 128, 475–482. doi: 10.1016/j.marpolbul.2018.01.051 29571399

[B30] Li W. Shi Y. Gao L. Liu J. Cai Y. (2012). Occurrence of antibiotics in water, sediments, aquatic plants, and animals from baiyangdian lake in north China. Chemosphere 89, 1307–1315. doi: 10.1016/j.chemosphere.2012.05.079 22698376

[B31] Liu S. Dong G. Zhao H. Chen M. Quan W. Qu B. (2018). Occurrence and risk assessment of fluoroquinolones and tetracyclines in cultured fish from a coastal region of northern China. Environ. Sci. pollut. R. 25, 8035–8043. doi: 10.1007/s11356-017-1177-6 29305805

[B32] Liu X. Steele J. C. Meng X. (2017b). Usage, residue, and human health risk of antibiotics in Chinese aquaculture: a review. Environ. pollut. 223, 161–169. doi: 10.1016/j.envpol.2017.01.003 28131482

[B33] Liu Y. Yang H. Yang S. Hu Q. Cheng H. Liu H.. (2013). High-performance liquid chromatography using pressurized liquid extraction for the determination of seven tetracyclines in egg, fish and shrimp. J. Chromatogr. B. 917, 11–17. doi: 10.1016/j.jchromb.2012.12.036 23353938

[B34] Liu Y. Zhang G. Sun R. Zhou S. Dong J. Yang Y.. (2022). Determination of pharmacokinetic parameters and tissue distribution characters of enrofloxacin and its metabolite ciprofloxacin in *Procambarus clarkii* after two routes of administration. Aquacult. Rep. 22, 100939. doi: 10.1016/j.aqrep.2021.100939

[B35] Liu S. Zhao H. Lehmler H. Cai X. Chen J. (2017a). Antibiotic pollution in marine food webs in laizhou bay, north China: trophodynamics and human exposure implication. Environ. Sci. Technol. 51, 2392–2400. doi: 10.1021/acs.est.6b04556 28106989 PMC5618103

[B36] Lu S. Lin C. Lei K. Wang B. Xin M. Gu X.. (2020). Occurrence, spatiotemporal variation, and ecological risk of antibiotics in the water of the semi-enclosed urbanized jiaozhou bay in eastern China. Water Res. 184, 116187. doi: 10.1016/j.watres.2020.116187 32707308

[B37] Lu Y. Jacobson D. Bousvaros A.. (2009). Immunizations in patients with inflammatory bowel disease. Inflamm. Bowel Dis. 15:1417–1423. doi: 10.1002/ibd.20941 PMC6637957 19408335

[B38] Ma D. Deng G. Bai J. Li S. Yu L. Quan Y.. (2013). A strain of *Siniperca chuatsi* rhabdovirus causes high mortality among cultured largemouth bass in south China. J. Aquat. Anim. Health 25, 197–204. doi: 10.1080/08997659.2013.799613 23915177

[B39] Mallory T. G. (2013). China's distant water fishing industry: Evolving policies and implications. Mar. Policy 38, 99–108. doi: 10.1016/j.marpol.2012.05.024

[B40] Menkem Z. Ngangom B. L. Tamunjoh S. S. A. Boyom F. F. (2019). Antibiotic residues in food animals: public health concern. Acta Ecol. Sin. 39, 411–415. doi: 10.1016/j.chnaes.2018.10.004

[B41] Ministry of Agriculture (2007). Specification for the application of sulfonamides in aquaculture (SC/T 1083-2007) (Beijing: China Agriculture Press), 1–5.

[B42] Ministry of Agriculture (2019). National food safety standard maximum residue limits for veterinary drugs in foods (GB 31650-2019) (Beijing, China: Ministry of Agriculture of the People's Republic of China).

[B43] Mishra S. P. (2018). Impact of high-pressure processing on chemical constituents and nutritional properties in aquatic foods: a review. Int. J. Food Sci. Tech. 53, 873–891. doi: 10.1111/ijfs.13693

[B44] Ni L. Chen D. Fu H. Xie Q. Lu Y. Wang X.. (2021). Residual levels of antimicrobial agents and heavy metals in 41 species of commonly consumed aquatic products in shanghai, China, and cumulative exposure risk to children and teenagers. Food Contr. 129, 108225. doi: 10.1016/j.foodcont.2021.108225

[B45] Norton B. L. Holland D. P. (2012). Current management options for latent tuberculosis: a review. Infect. Drug Resist. 5, 163–173. doi: 10.2147/IDR.S29180 23226700 PMC3514970

[B46] Olivares-Fuster O. Arias C. R. (2011). Development and characterization of rifampicin-resistant mutants from high virulent strains of flavobacterium columnare. J. Fish. Dis. 34, 385–394. doi: 10.1111/j.1365-2761.2011.01253.x 21488906

[B47] Postigo C. Richardson S. D. (2014). Transformation of pharmaceuticals during oxidation/disinfection processes in drinking water treatment. J. Hazard. Mater. 279, 461–475. doi: 10.1016/j.jhazmat.2014.07.029 25156529

[B48] Rico A. Satapornvanit K. Haque M. M. Min J. Nguyen P. T. Telfer T. C.. (2012). Use of chemicals and biological products in Asian aquaculture and their potential environmental risks: a critical review. Rev. Aquacult. 4, 75–93. doi: 10.1111/j.1753-5131.2012.01062.x

[B49] Seung K. J. Gelmanova I. E. Peremitin G. G. Golubchikova V. T. Pavlova V. E. Sirotkina O. B.. (2004). The effect of initial drug resistance on treatment response and acquired drug resistance during standardized short-course chemotherapy for tuberculosis. Clin. Infect. Dis. 39, 1321–1328. doi: 10.1086/425005 15494909

[B50] Song C. Zhang C. Kamira B. Qiu L. Fan L. Wu W.. (2017). Occurrence and human dietary assessment of sulfonamide antibiotics in cultured fish around tai lake, China. Environ. Toxicol. Chem. 36, 2899–2905. doi: 10.1007/s11356-017-9442-2 28585696

[B51] Urbano V. R. Maniero M. G. Pérez-Moya M. Guimarães J. R. (2017). Influence of pH and ozone dose on sulfaquinoxaline ozonation. J. Environ. Manage. 195, 224–231. doi: 10.1016/j.jenvman.2016.08.019 27558831

[B52] Varol M. Sünbül M. R. (2019). Environmental contaminants in fish species from a large dam reservoir and their potential risks to human health. Ecotox. Environ. Safe. 169, 507–515. doi: 10.1016/j.ecoenv.2018.11.060 30472475

[B53] Wang M. Helbling D. E. (2016). A non-target approach to identify disinfection byproductsof structurally similar sulfonamide antibiotics. Water Res. 102, 241–251. doi: 10.1016/j.watres.2016.06.042 27348196

[B54] Wang A. Ran C. Wang Y. Zhang Z. Ding Q. Yang Y.. (2019). Use of probiotics in aquaculture of China-a review of the past decade. Fish. Shellfish. Immunol. 86, 734–755. doi: 10.1016/j.fsi.2018.12.026 30553887

[B55] Wang H. Wang N. Wang B. Fang H. Fu C. Tang C.. (2016). Antibiotics detected in urines and adipogenesis in school children. Environ. Int. 89, 204–211. doi: 10.1016/j.envint.2016.02.005 26882350

[B56] Xu Y. Guo C. Lv J. Hou S. Luo Y. Zhang Y.. (2018). Spatiotemporal profile of tetracycline and sulfonamide and their resistance on a catchment scale. Environ. pollut. 241, 1098–1105. doi: 10.1016/j.envpol.2018.06.050 30029318

[B57] Xu W. Zhu X. Wang X. Deng L. Zhang G. (2006). Residues of enrofloxacin, furazolidone and their metabolites in Nile tilapia (*Oreochromis niloticus*). Aquaculture 254, 1–8. doi: 10.1016/j.aquaculture.2005.10.030

[B58] Ye C. Shi J. Zhang X. Qin L. Jiang Z. Wang J.. (2021). Occurrence and bioaccumulation of sulfonamide antibiotics in different fish species from hangbu-fengle river, southeast China. Environ. Sci. pollut. R. 28, 44111–44123. doi: 10.1007/s11356-021-13850-5 33842998

[B59] Zaki S. A. Shanbag P. Bhongade S. (2013). Red man syndrome due to accidental overdose of rifampicin. Indian J. Crit. Care M. 17, 55–56. doi: 10.4103/0972-5229.112152 23833481 PMC3701402

[B60] Zeng X. Zhang L. Chen Q. Yu K. Zhao S. Zhang L.. (2020). Maternal antibiotic concentrations in pregnant women in shanghai and their determinants: a biomonitoring-based prospective study. Environ. Int. 138, 105638. doi: 10.1016/j.envint.2020.105638 32179319

[B61] Zhang X. Zhang J. Han Q. Wang X. Wang S. Yuan X.. (2021). Antibiotics in mariculture organisms of different growth stages: tissue-specific bioaccumulation and influencing factors. Environ. pollut. 288, 117715. doi: 10.1016/j.envpol.2021.117715 34256288

[B62] Zhang R. Zhang G. Zheng Q. Tang J. Chen Y. Xu W.. (2012). Occurrence and risks of antibiotics in the laizhou bay, China: impacts of river discharge. Ecotox. Environ. Safe. 80, 208–215. doi: 10.1016/j.ecoenv.2012.03.002 22444724

[B63] Zhang J. Zhang X. Zhou Y. Han Q. Wang X. Song C.. (2023). Occurrence, distribution and risk assessment of antibiotics at various aquaculture stages in typical aquaculture areas surrounding the yellow Sea. J. Environ. Sci. 126, 621–632. doi: 10.1016/j.jes.2022.01.024 36503788

[B64] Zhao L. Dong Y. H. Wang H. (2010). Residues of veterinary antibiotics in manures from feedlot livestock in eight provinces of China. Sci. Tot. Environ. 408, 1069–1075. doi: 10.1016/j.scitotenv.2009.11.014 19954821

[B65] Zhou L. Wang W. Lv Y. Mao Z. Chen C. Wu Q. L. (2020). Tissue concentrations, trophic transfer and human risks of antibiotics in freshwater food web in lake taihu, China. Ecotox. Environ. Safe. 197, 110626. doi: 10.1016/j.ecoenv.2020.110626 32339959

[B66] Zhou L. Ying G. Liu S. Zhao J. Chen F. Zhang R.. (2012). Simultaneous determination of human and veterinary antibiotics in various environmental matrices by rapid resolution liquid chromatography-electrospray ionization tandem mass spectrometry. J. Chromatogr. A. 1244, 123–138. doi: 10.1016/j.chroma.2012.04.076 22625208

[B67] Zhou Y. Zhu F. Zheng D. Gao M. Guo B. Zhang N.. (2021). Detection of antibiotics in the urine of children and pregnant women in jiangsu, China. Environ. Res. 196, 110945. doi: 10.1016/j.envres.2021.110945 33647296

