# Supplementary material for: Occurrence and human health risk assessment of antibiotics in cultured fish from 19 provinces in China
Source: Front Cell Infect Microbiol. 2022 Aug 2;12:964283. doi: 10.3389/fcimb.2022.964283 (PMC9378958; doi:10.3389/fcimb.2022.964283)
Supplement: Supplementary file 1 [file DataSheet_1.docx]

Supplementary Material

## Figures


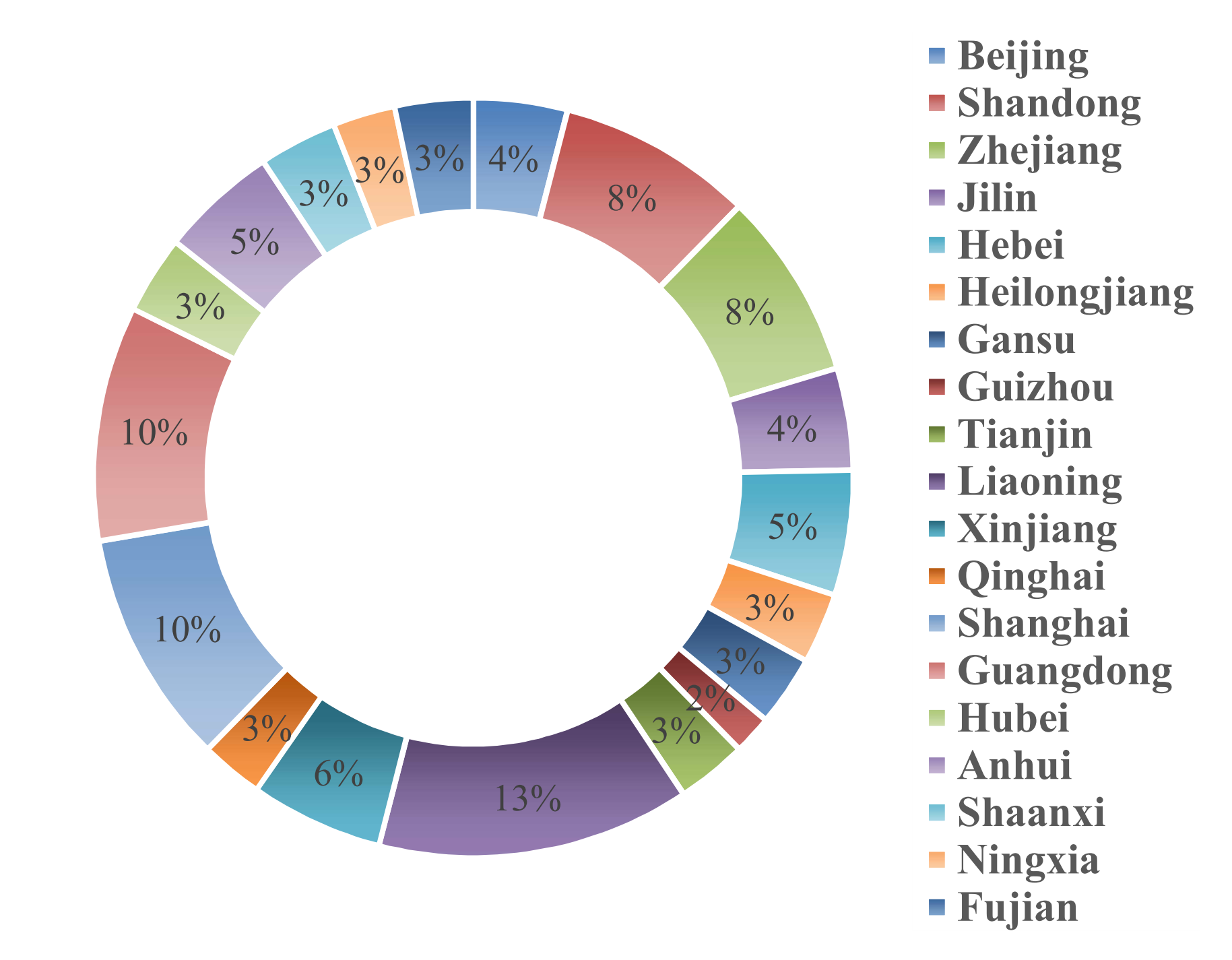


**Figure S1.** The proportion of the samples in different provinces.


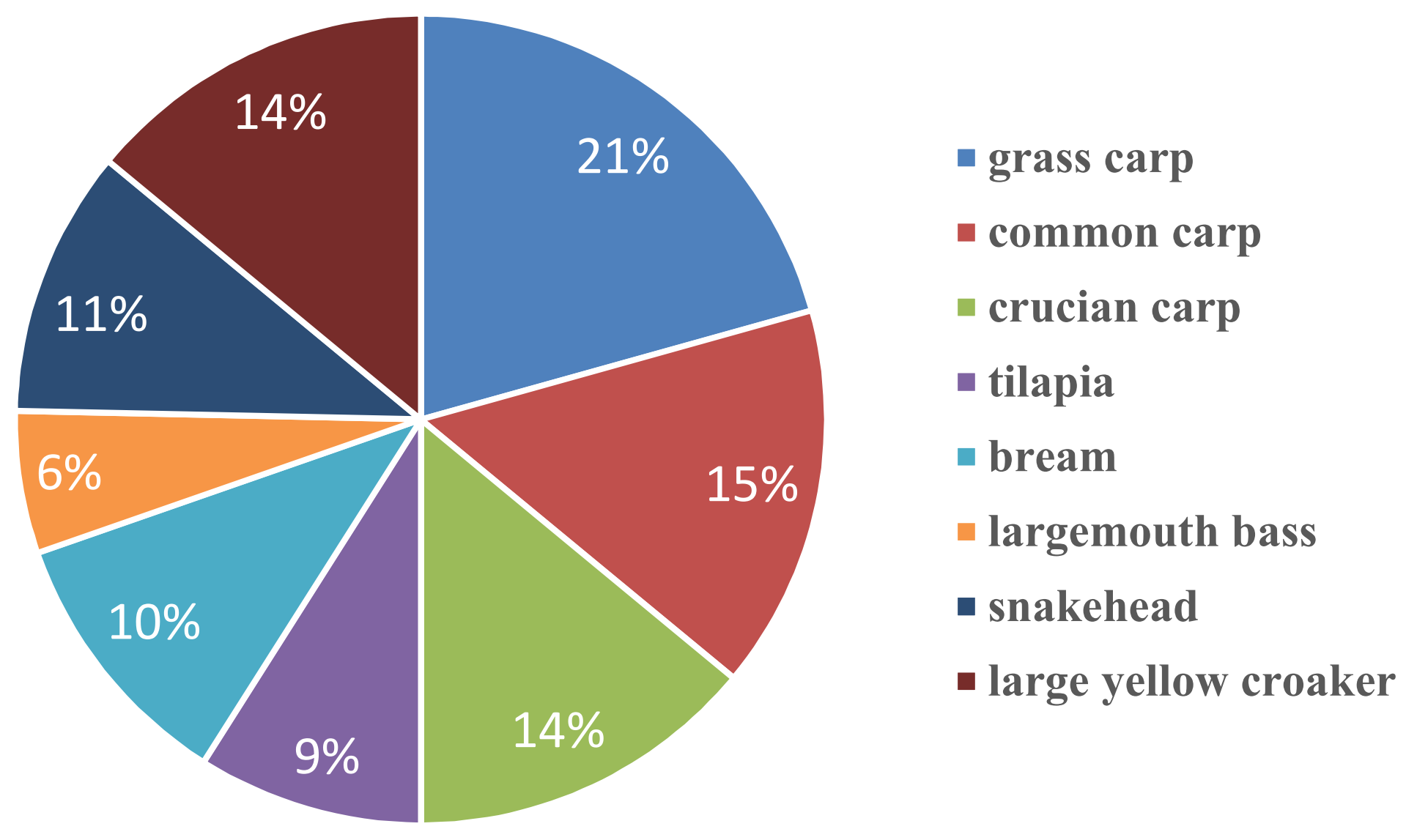


**Figure S2.** The proportion of the samples in different fish species.





**Figure S3.** Frequency distribution of enrofloxacin, ciprofloxacin and enoxacin in the positive samples.





**Figure S4.** Concentrations of antibiotics in different aquatic species.





**Figure S5.** Detection frequency distribution of total antibiotics in different province. The province with no positive detection was not presented in this figure.

**Tables**

**Table S1** Details of the 8 macrolides, 4 tetracyclines, 15 fluoroquinolones and rifampin standard antibiotic in the study.

| Serial number | Antibiotics | CAS | LOD  (μg/kg) | LOQ  (μg/kg) | ADI  (μg/kg.bw) |
| --- | --- | --- | --- | --- | --- |
| 1 | Kitasamycin | 1392-21-8 | 1.0 | 4.0 |  |
| 2 | Spiramycin | 8025-81-8 |  |  |  |
| 3 | Tilmicosin | 108050-54-0 |  |  |  |
| 4 | Tylosin | 1401-69-0 |  |  |  |
| 5 | Clarithromycin | 18323-44-9 |  |  |  |
| 6 | Oleandomycin | 7060-74-4 |  |  |  |
| 7 | Erythromycin | 114-07-8 |  |  | 5 |
| 8 | Azithromycin | 83905-01-5 |  |  |  |
| 9 | Tetracycline | 60-54-8 | 20.0 | 50.0 |  |
| 10 | Oxytetracycline | 79-57-2 |  |  |  |
| 11 | Chlortetracycline | 57-62-5 |  |  |  |
| 12 | Doxycycline | 564-25-0 |  |  | 5 |
| 13 | Difloxacin | 98106-17-3 | 1.0 | 2.0 |  |
| 14 | Sparfloxacin | 110871-86-8 |  |  |  |
| 15 | Norfloxacin | 70458-96-7 |  |  |  |
| 16 | Danofloxacin | 112398-08-0 |  |  |  |
| 17 | Fleroxacin | 79660-72-3 |  |  |  |
| 18 | Flumequine | 42835-25-6 |  |  |  |
| 19 | Ofloxacin | 82419-36-1 |  |  |  |
| 20 | Pefloxacin | 70458-92-3 |  |  |  |
| 21 | Ciprofloxacin | 85721-33-1 |  |  | 2 |
| 22 | Enrofloxacin | 93106-60-6 |  |  | 2 |
| 23 | Lomefloxacin | 98079-51-7 |  |  |  |
| 24 | Orbifloxacin | 113617-63-3 |  |  |  |
| 25 | Sarafloxacin | 98105-99-8 |  |  |  |
| 26 | Oxolinic Acid | 14698-29-4 |  |  |  |
| 27 | Enoxacin | 74011-58-8 |  |  |  |
| 28 | Rifampin | 13292-46-1 | 1.0 | 3.0 | 10000 |

**Table S2** Liquid phase gradient elution procedure. A: water with 0.05% formic acid; B: 50% acetonitrile 50% methanol 5% water with 0.05% formic acid.

| Time (min) | Flow (mL/min) | A (%) | B (%) |
| --- | --- | --- | --- |
| 0.0 | 0.3 | 98 | 2 |
| 2.0 | 0.3 | 98 | 2 |
| 3.0 | 0.3 | 80 | 20 |
| 11.0 | 0.3 | 0 | 100 |
| 14.0 | 0.3 | 0 | 100 |
| 14.5 | 0.3 | 98 | 2 |
| 16.0 | 0.3 | 98 | 2 |

**Table S3** Optimal MS/MS spectrometric conditions of the 28 antibiotics.

| Antibiotics | Parent (m/z) | Daughter (m/z) | Collision (V) |
| --- | --- | --- | --- |
| Kitasamycin | 772 | 109 | 38 |
|  |  | 174* | 30 |
| Spiramycin | 843 | 142 | 40 |
|  |  | 174* | 36 |
| Tilmicosin | 870 | 138 | 41 |
|  |  | 696* | 36 |
| Tylosin | 916 | 772 | 29 |
|  |  | 174* | 36 |
| Clarithromycin | 749 | 590 | 18 |
|  |  | 158* | 28 |
| Oleandomycin | 688 | 158 | 28 |
|  |  | 544* | 16 |
| Erythromycin | 734 | 158 | 28 |
|  |  | 576* | 18 |
| Azithromycin | 750 | 591 | 27 |
|  |  | 158* | 36 |
| Tetracycline | 445 | 427 | 19 |
|  |  | 410* | 29 |
| Oxytetracycline | 461 | 426 | 27 |
|  |  | 443* | 21 |
| Chlortetracycline | 479 | 462 | 27 |
|  |  | 444* | 33 |
| Doxycycline | 445 | 154 | 37 |
|  |  | 428* | 29 |
| Difloxacin | 400 | 299 | 28 |
|  |  | 356* | 19 |
| Sparfloxacin | 393 | 251 | 32 |
|  |  | 292* | 24 |
| Norfloxacin | 320 | 233 | 24 |
|  |  | 276* | 16 |
| Danofloxacin | 358 | 283 | 22 |
|  |  | 340 | 22 |
| Fleroxacin | 370 | 269 | 26 |
|  |  | 326* | 19 |
| Flumequine | 262 | 202 | 32 |
|  |  | 244 | 18 |
| Ofloxacin | 362 | 58 | 34 |
|  |  | 261* | 30 |
| Pefloxacin | 334 | 42 | 58 |
|  |  | 70* | 40 |
| Ciprofloxacin | 332 | 231 | 30 |
|  |  | 245* | 22 |
| Enrofloxacin | 360 | 72 | 30 |
|  |  | 84* | 30 |
| Lomefloxacin | 352 | 44 | 38 |
|  |  | 265* | 22 |
| Orbifloxacin | 396 | 41 | 40 |
|  |  | 295* | 26 |
| Sarafloxacin | 386 | 255 | 50 |
|  |  | 299* | 24 |
| Oxolinic Acid | 262 | 201 | 28 |
|  |  | 215* | 28 |
| Enoxacin | 321 | 232 | 34 |
|  |  | 303* | 21 |
| Rifampin | 823 | 399 | 35 |
|  |  | 791* | 23 |

* As quantitative ion

**Table S4** Mean concentration (µg/kg) of antibiotics in relation to selected characteristics.

| Antibiotics | Overall | grass carp | common carp | crucian carp | tilapia | bream | largemouth bass | Snakehead | Large yellow croaker |
| --- | --- | --- | --- | --- | --- | --- | --- | --- | --- |
| Oleandomycin | 0.58 | - | - | - | - | - | - | - | 1.09 |
| Erythromycin | 1.18 | - | - | - | - | - | - | - | 5.39 |
| Azithromycin | 0.64 | - | - | - | - | - | 2.89 | - | - |
| **Macrolides**^a^ | **4.90** | **4.0** | **4.0** | **4.0** | **4.0** | **4.0** | **6.39** | **4.0** | **9.48** |
| Oxytetracycline | 10.1 | - | - | - | - | - | - | - | 10.54 |
| Chlortetracycline | 10.9 | - | 13.74 | 11.09 | 12.01 | - | - | - | - |
| Doxycycline | 13.4 | 16.45 | 19.64 | 11.47 | - | 12.17 | - | 11.20 | - |
| **Tetracyclines**^a^ | **44.36** | **46.45** | **53.38** | **42.56** | **42.01** | **42.17** | **40.00** | **41.20** | **40.54** |
| Ciprofloxacin | 0.69 | - | 0.59 | - | - | 0.63 | - | 1.98 | - |
| Enrofloxacin | 2.50 | 2.91 | 3.44 | 0.78 | 6.59 | 1.42 | 1.31 | 3.04 | 0.84 |
| Enoxacin | 0.52 | - | 0.56 | - | - | - | - | 0.59 | - |
| **Fuoroquinolones**^a^ | **9.70** | **9.91** | **10.59** | **7.78** | **13.59** | **8.55** | **8.31** | **11.61** | **7.84** |
| Rifampin | 0.53 | 0.576 | 0.50 | 0.50 | 0.50 | 0.67 | 0.50 | 0.50 | 0.50 |
| **Total**^b^ |  | **60.94** | **68.47** | **54.84** | **60.10** | **55.39** | **55.20** | **57.31** | **58.36** |

^a^ Mean concentrations of antibiotics in corresponding class for individual. Antibiotic concentrations below LOD were replaced with those of LOD/2.

^b^ Sum of concentrations of all antibiotics.

**Table S5** Mean concentration (µg/kg) of antibiotics in relation to selected characteristics.

| Sites^b^ | Tatol | Macrolides^a^ | Tetracyclines^a^ | Fuoroquinolones^a^ | Rifampicin^a^ |
| --- | --- | --- | --- | --- | --- |
| Beijing | 64.4 | 4.0 | 51.6 | 8.37 | 0.5 |
| Shandong | 88.2 | 4.0 | 76.2 | 7.50 | 0.5 |
| Zhejiang | 53.3 | 4.0 | 40.9 | 7.87 | 0.5 |
| Jilin | 58.0 | 4.0 | 40.0 | 13.5 | 0.5 |
| Hebei | 52.9 | 4.0 | 40.0 | 8.35 | 0.5 |
| Heilongjiang | 83.5 | 4.0 | 59.7 | 19.3 | 0.5 |
| Gansu | 57.6 | 4.0 | 40.0 | 13.1 | 0.5 |
| Guizhou | 59.6 | 4.0 | 46.0 | 9.09 | 0.5 |
| Tianjin | 52.6 | 4.0 | 40.0 | 8.07 | 0.5 |
| Liaoning | 52.7 | 4.0 | 40.0 | 8.19 | 0.5 |
| Xinjiang | 55.1 | 4.0 | 40.0 | 10.3 | 0.82 |
| Qinghai | 74.7 | 9.09 | 40.0 | 25.1 | 0.5 |
| Shanghai | 52.3 | 4.0 | 40.3 | 7.50 | 0.5 |
| Guangdong | 66.0 | 11.7 | 40.8 | 12.8 | 0.66 |
| Hubei | 52.9 | 4.0 | 40.0 | 8.36 | 0.5 |

^a^ Mean concentrations of antibiotics in corresponding class. Antibiotic concentrations below LOD were replaced with those of LOD/2.

^b^ Sum of concentrations of all antibiotics.

**Table S6** Estimated daily exposure dose (μg/kg/day) of 28 antibiotics per fish species.

| Antibiotics | Overall | grass carp | common carp | crucian carp | tilapia | bream | largemouth bass | Snakehead | Large yellow croaker |
| --- | --- | --- | --- | --- | --- | --- | --- | --- | --- |
| Oleandomycin | 0.00054 | - | - | - | - | - | - | - | 0.0010 |
| Erythromycin | 0.0011 | - | - | - | - | - | - | - | 0.0050 |
| Azithromycin | 0.00060 | - | - | - | - | - | 0.0027 | - | - |
| **Macrolides**^a^ | **0.0046** | **0.0037** | **0.0037** | **0.0037** | **0.0037** | **0.0037** | **0.0060** | **0.0037** | **0.0088** |
| Oxytetracycline | 0.0094 | - | - | - | - | - | - | - | 0.0098 |
| Chlortetracycline | 0.010 | - | 0.013 | 0.010 | 0.011 | - | - | - | - |
| Doxycycline | 0.013 | 0.015 | 0.018 | 0.011 | - | 0.011 | - | 0.010 | - |
| **Tetracyclines**^a^ | **0.041** | **0.043** | **0.050** | **0.040** | **0.039** | **0.039** | **0.037** | **0.039** | **0.038** |
| Ciprofloxacin | 0.00064 | - | 0.00055 | - | - | 0.00059 | - | 0.0018 | - |
| Enrofloxacin | 0.0023 | 0.0027 | 0.0032 | 0.00073 | 0.0062 | 0.0013 | 0.0012 | 0.0028 | 0.00079 |
| Enoxacin | 0.00049 | - | 0.00053 | - | - | - | - | 0.00055 | - |
| **Fuoroquinolones**^a^ | **0.0091** | **0.0092** | **0.0099** | **0.0073** | **0.013** | **0.0080** | **0.0078** | **0.011** | **0.0073** |
| Rifampin | **0.00049** | 0.00054 | 0.00047 | 0.00047 | 0.00047 | 0.00063 | 0.00047 | 0.00047 | 0.00047 |
| **Total**^b^ | **0.055** | **0.057** | **0.064** | **0.051** | **0.056** | **0.052** | **0.052** | **0.053** | **0.054** |

^a^ Sum of estimated daily intakes of antibiotics in corresponding class for individual.

^b^ Sum of estimated daily intakes of all antibiotics; -, < limits of detection (LODs).

**Table S7** Estimated daily exposure dose (EDI) (μg/kg/day), hazard quotient (HQ) and hazard index (HI) of antibiotics per provinces.

| Sites | Total^b^ | | Macrolides^a^ | | Tetracyclines^a^ | | Fuoroquinolones^a^ | | Rifampicin^a^ | |
| --- | --- | --- | --- | --- | --- | --- | --- | --- | --- | --- |
|  | EDI | HI×10^-2^ | EDI | HQ×10^-2^ | EDI | HQ×10^-2^ | EDI | HQ×10^-2^ | EDI | HQ×10^-2^ |
| Beijing | 0.060 | 1.42 | 0.0037 | 0.074 | 0.048 | 0.96 | 0.0078 | 0.39 | 0.00047 | 4.7E-06 |
| Shandong | 0.082 | 1.84 | 0.0037 | 0.074 | 0.071 | 1.42 | 0.0070 | 0.35 | 0.00047 | 4.7E-06 |
| Zhejiang | 0.049 | 1.20 | 0.0037 | 0.074 | 0.038 | 0.76 | 0.0073 | 0.37 | 0.00047 | 4.7E-06 |
| Jilin | 0.054 | 1.46 | 0.0037 | 0.074 | 0.037 | 0.74 | 0.013 | 0.65 | 0.00047 | 4.7E-06 |
| Hebei | 0.049 | 1.20 | 0.0037 | 0.074 | 0.037 | 0.74 | 0.0078 | 0.39 | 0.00047 | 4.7E-06 |
| Heilongjiang | 0.078 | 2.09 | 0.0037 | 0.074 | 0.056 | 1.12 | 0.018 | 0.90 | 0.00047 | 4.7E-06 |
| Gansu | 0.053 | 1.41 | 0.0037 | 0.074 | 0.037 | 0.74 | 0.012 | 0.60 | 0.00047 | 4.7E-06 |
| Guizhou | 0.056 | 1.36 | 0.0037 | 0.074 | 0.043 | 0.86 | 0.0085 | 0.43 | 0.00047 | 4.7E-06 |
| Tianjin | 0.049 | 1.19 | 0.0037 | 0.074 | 0.037 | 0.74 | 0.0075 | 0.38 | 0.00047 | 4.7E-06 |
| Liaoning | 0.049 | 1.19 | 0.0037 | 0.074 | 0.037 | 0.74 | 0.0076 | 0.38 | 0.00047 | 4.7E-06 |
| Xinjiang | 0.051 | 1.29 | 0.0037 | 0.074 | 0.037 | 0.74 | 0.0096 | 0.48 | 0.00077 | 7.7E-06 |
| Qinghai | 0.069 | 2.06 | 0.0085 | 0.17 | 0.037 | 0.74 | 0.023 | 1.20 | 0.00047 | 4.7E-06 |
| Shanghai | 0.049 | 1.18 | 0.0037 | 0.074 | 0.038 | 0.76 | 0.0070 | 0.35 | 0.00047 | 4.7E-06 |
| Guangdong | 0.062 | 1.58 | 0.011 | 0.22 | 0.038 | 0.76 | 0.012 | 0.60 | 0.00061 | 6.1E-06 |
| Hubei | 0.049 | 1.20 | 0.0037 | 0.074 | 0.037 | 0.74 | 0.0078 | 0.39 | 0.00047 | 4.7E-06 |

^a^ Sum of estimated daily intakes and hazard quotient in corresponding class for individual.

^b^ Sum of estimated daily intakes and hazard index of all antibiotics.
